# Selective Detection of *Legionella pneumophila* Serogroup 1 and 5 with a Digital Photocorrosion Biosensor Using Antimicrobial Peptide-Antibody Sandwich Strategy

**DOI:** 10.3390/bios12020105

**Published:** 2022-02-09

**Authors:** M. Amirul Islam, Walid M. Hassen, Ishika Ishika, Azam F. Tayabali, Jan J. Dubowski

**Affiliations:** 1 Laboratory for Quantum Semiconductors and Photon-Based BioNanotechnology, Interdisciplinary Institute for Technological Innovation (3IT), CNRS UMI-3463, Department of Electrical and Computer Engineering, Université de Sherbrooke, 3000, Boul. de l’Université, Sherbrooke, QC J1K 0A5, Canada; mohammed.amirul.islam@usherbrooke.ca (M.A.I.); mohamed.walid.hassen@usherbrooke.ca (W.M.H.); ishika.ishika@usherbrooke.ca (I.I.); 2 Biotechnology Laboratory, Environmental Health Science and Research Bureau, Healthy Environments and Consumer Safety Branch, Environmental Health Centre, Health Canada, Ottawa, ON K1A 0K9, Canada; azam.tayabali@hc-sc.gc.ca

**Keywords:** cysteine-modified warnericin RK, antimicrobial peptides, anti-*Legionella pneumophila* polyclonal antibody, digital photocorrosion biosensor, GaAs/AlGaAs nanoheterostructures

## Abstract

Rapid detection of *Legionella pneumophila* (*L. pneumophila*) is important for monitoring the presence of these bacteria in water sources and preventing the transmission of the Legionnaires’ disease. We report improved biosensing of *L. pneumophila* with a digital photocorrosion (DIP) biosensor functionalized with an innovative structure of cysteine-modified warnericin antimicrobial peptides for capturing bacteria that are subsequently decorated with anti-*L. pneumophila* polyclonal antibodies (pAbs). The application of peptides for the operation of a biosensing device was enabled by the higher bacterial-capture efficiency of peptides compared to other traditional ligands, such as those based on antibodies or aptamers. At the same time, the significantly stronger affinity of pAbs decorating the *L. pneumophila* serogroup-1 (SG-1) compared to serogroup-5 (SG-5) allowed for the selective detection of *L. pneumophila* SG-1 at 50 CFU/mL. The results suggest that the attractive sensitivity of the investigated sandwich method is related to the flow of an extra electric charge between the pAb and a charge-sensing DIP biosensor. The method has the potential to offer highly specific and sensitive detection of *L. pneumophila* as well as other pathogenic bacteria and viruses.

## 1. Introduction

Rapid detection of pathogenic *Legionella pneumophila* (*L. pneumophila*) in water environments is a key challenge in preventing related illness outbreaks [1,2]. Presently, culture-based methods are widely used and considered gold standard techniques for detecting pathogenic *L. pneumophila* [3]. However, these approaches are both labor intensive and time consuming [4], typically taking up to ~10 days to quantify growing bacterial colonies [5]. Other techniques, such as polymerase chain reaction (PCR) and matrix-assisted laser desorption/ionization time-of-flight (MALDI-TOF) spectroscopy provide accurate and relatively fast detection [6]. However, the requirement of highly trained personnel and sophisticated laboratory equipment are the main constraints for wide application of these techniques [7]. Therefore, research interests have been directed to avail cost effective, fast, portable, and less labor-intensive tools for detecting *L. pneumophila* [2,8,9].

Numerous immunosensors investigated for the detection of *L. pneumophila* are listed in Table 1. These sensors undoubtedly offer specific and rapid detection of bacteria; however, the operation of most of them is restricted to laboratory settings due to the need for instrumentation that is not suitable for field applications and sophisticated stepwise biochemical protocols. For instance, Park, et al. [10] have reported a DNA biosensor for the specific detection of *L. pneumophila*, but the extraction of DNA from bacteria is associated with a number of processing steps, resulting in laborious and costly analysis. Whole cell *L. pneumophila* biosensors have frequently been investigated based on electrochemical impedance spectroscopy (EIS) [11,12], surface plasmon resonance (SPR) [13], and colorimetric detection [14,15], as presented in Table 1. EIS biosensors have received significant attention due to their sensitivity and cost effectiveness [16]. However, the drifting of the electrochemical signal related to changes of buffer chemistry affects the performance and reproducibility of such devices [17]. SPR biosensors have some advantages related to label-free detection, sensitivity, and applicability to real-time kinetic measurements [18]. However, SPR biosensors are also sensitive to temperature variations and require special temperature-stabilized chambers [19]. Colorimetric paper-based biosensors [15,20] are potentially attractive due to their ability to monitor the presence of specific pathogens by detecting change in colors distinguishable with a naked eye. However, the major limitation of colorimetric assays is their low sensitivity since it is often difficult to transform biochemical reactions into measurable color changes [20]. An innovative biosensing method based on digital photocorrosion (DIP) of GaAs/AlGaAs semiconductor nanoheterostructures has been recently introduced for rapid detection of *L. pneumophila* [21,22]. The method is sensitive to charge transfer between semiconductor and immobilized biomolecules, and decorating *L. pneumophila* with negatively charged sodium dodecyl sulfate (SDS) permitted detection at 10^3^ CFU/mL with polyclonal antibody (pAb) functionalized DIP biochips [23].

The functioning of *L. pneumophila* biosensors have frequently been based on the application of antibodies (Abs) as bacteria-recognizing ligands [11,27]. The limitation of this approach is largely related to the dependency on animal-based production, which is prone to batch-to-batch variations [28]. Furthermore, the interaction of bacteria with Abs favors free liquid space (3D method) compared to the interaction with Abs immobilized on a biochip surface (2D method) [29], while the orientation of Abs immobilized on the surface might also influence the capture of bacteria [30,31]. There also has been growing interest in exploring antimicrobial peptides (AMPs) as binding moieties designed for capturing bacteria on biosensor surfaces [32,33]. The AMP ligands can be obtained by employing synthetic processes [34,35,36]; some cationic AMPs maintain strong affinity even after exposure to extreme environmental conditions, such as autoclaving and boiling [32,37]. Thus, the increased stability of AMPs in comparison to typical globular proteins, such as Abs, is potentially advantageous for biosensing applications [32,38]. We have explored warnericin RK AMP for the application in a DIP biosensor and demonstrated rapid detection of *L. pneumophila* at 10^3^ CFU/mL [39]. In a follow up publication [40], we reported that a cysteine-modified warnericin RK AMP (Cys RK AMP) biosensing architecture increased sensitivity to 200 CFU/mL.

However, the relatively broad specificity spectrum of AMP towards different bacteria raised the question about the specificity of the proposed biosensor, especially for detection of the *L. pneumophila* serogroup 1 (SG1) that is responsible for over 85% of *L. pneumophila* related disease outbreaks [41]. To address this question, we have investigated the sandwich approach involving pAbs for decorating captured *L. pneumophila*. We have also verified that the applied pAb exhibited four times greater capture efficiency of *L. pneumophila* SG1 than that of *L. pneumophila* SG5 and confirmed with the DIP-biosensing results reported in this work.

## 2. Materials and Methods

### 2.1. Materials and Reagents

The chips (2 mm × 2 mm) were cut from a 5 cm diameter wafer comprising a stack of GaAs and Al_0.35_Ga_0.65_As layers grown on the GaAs (001) substrate (CMC Microelectronics, Kingston, ON, Canada). More details about this wafer and the mechanisms of digital photocorrosion of GaAs/AlGaAs nanoheterostructures were published somewhere else [42,43]. The application of a GaAs/Al_0.35_Ga_0.65_As nanoheterostructure for building DIP biosensors was discussed earlier [21,43,44,45]. The results reported in this paper were obtained by recording DIP of the topmost pair of GaAs (12 nm thick) and AlGaAs (10 nm thick) layers.

Undoped double-sided polished GaAs (001) wafer (WV 23084) purchased from Wafer Technology Ltd. (Payson, AZ, USA) was used to investigate biofunctionalization and to evaluate the bacteria-capture efficiency.

High-quality (semiconductor grade) acetone, isopropanol, anhydrous ethanol, and OptiClear were purchased, from National Diagnostics (Mississauga, ON, Canada), Fisher Scientific (Ottawa, ON, Canada), and ACP (Montréal, QC, Canada), respectively. The 28% ammonium hydroxide (NH4OH) was purchased from Anachemia (Richmond, BC, Canada). Phosphate-buffered saline (PBS; pH 7.4) at 10×, Luria-Bertani (LB) medium, isopropyl thio-β-galacto-side (IPTG), and chloramphenicol were all purchased from Sigma-Aldrich (Oakville, ON, Canada). BCYE agar medium was obtained from VWR (Ontario, ON, Canada). Polyclonal Abs (anti-L. pneumophila) were purchased from ViroStat, Inc., catalog number 6051 prepared against the L. pneumophila SG1, ATCC 33152. The green fluorescent (GFP) L. pneumophila JR32 was kindly donated by Pr. Faucher from the Faculty of Agricultural and Environ-mental Sciences, McGill University (Ste-Anne de Bellevue, QC, Canada). Green fluo-rescent Escherichia coli K12 BW25113 (GFP E. coli) was obtained from the Department of Microbiology and Infectiology of the Université de Sherbrooke (UdS) Faculty of Medicine (Sherbrooke, QC, Canada), Bacillus subtilis ATCC 6051 (B. subtilis) was obtained from the Department of Biology of the UdS Faculty of Sciences (Sherbrooke, QC, Canada), and Pseudomonas fluorescens ATCC 13525 (P. fluorescens) was purchased from Cedarlane (Burlington, ON, Canada). The follow-ing, cysteine-modified AMPs: Cysteine-warnericin (Cys-WRK), Cysteine phenol-soluble modulins (Cys-PSM), and Cysteine-H2U (Cys-H2U) were synthesized by GenScript, Pis-cataway, USA and employed for the functionalization of GaAs or GaAs/AlGaAs chips targeting L. pneumophila.

### 2.2. Biofunctionalization of GaAs Chips

**Figure 1:**
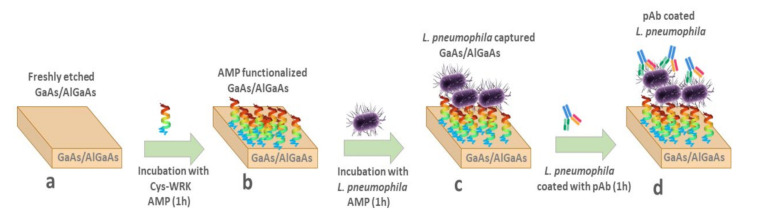
Schematic diagram of biosensor development, (**a**) freshly etched GaAs/AlGaAs nanoheterostructures; (**b**) adsorption of thiolated AMPs on GaAs/AlGaAs; (**c**) immobilization of bacteria on AMP-functionalized GaAs/AlGaAs; (**d**) immobilization of anti-*L. pneumophila* pAb on the surface of bacteria.

The 2 mm × 2 mm GaAs chips (bulk or GaAs/AlGaAs nanoheterostructures) were cleaned by successive dipping in acetone, OptiClear, and isopropanol for 5 min under ultrasonication and then dried with highly pure compressed nitrogen gas [46,47]. Thereafter, native oxides present on the surface of samples were removed by immersion in 28% NH_4_OH for 2 min at room temperature, followed by rinsing with degassed ethanol and subsequently degassed deionized (DI) water. Then, individual samples were incubated in each of the thiolated AMP solutions (50 µg/mL) for 1 h to allow for the Cys-AMPs attachment to the GaAs surface through the formation of covalent bonds between Ga or As atoms and the cysteine sulfur (S). The functionalized chips were sonicated in degassed DI water for 1 min and immediately rinsed with degassed DI water to remove noncovalently bound peptides and subsequently incubated in bacterial suspensions for 1h. Bacterial-bound chips were rinsed with DI water to remove unbound or loosely bound bacteria. The bacteria decoration step with anti-*L. pneumophila* pAb was completed by incubation with 100 μg/mL anti-*L. pneumophila* pAb for 30 min. This concentration of pAb is considered sufficient to saturate bacteria in a reproducible fashion. The procedure of biochip preparation is schematically illustrated in Figure 1.

### 2.3. Preparation of Bacterial Suspensions

Cultures of *P. fluorescens*, *E. coli*, and *B. subtilis* were grown in Luria-Bertani (LB) medium. Cultures of green fluorescent *L. pneumophila* SG1 and nonfluorescent SG5 were grown in buffered charcoal yeast extract agar (BCYE) medium with L-Cysteine. For the SG1 strain, the medium was supplemented with isopropyl thio-β-galactoside (IPTG) to induce the production of the green fluorescent protein (GFP) and chloramphenicol to maintain the plasmid encoding for the GFP. After growth, a few colonies were placed in 0.1× PBS and concentrations were determined by optical density measurements at 600 nm (OD_600_ nm).

### 2.4. Capture Efficiency of L. pneumophila SG1 and SG5 with pAb-Functionalized GaAs

The pAbs were prepared against the whole cell of a *L. pneumophila* SG1 strain [48]. However, the cross-reactivity is expected from various *L. pneumophila* serogroups due to the polyclonal character of these Abs. To evaluate the affinity of the used pAbs against the *L. pneumophila* SG1 and SG5, freshly cleaned and oxide-etched GaAs chips were functionalized for 20 h using a 1 mM of mercaptohexadecanoic acid (MHDA) solution in degassed ethanol. To capture Abs, the samples were incubated for 30 min in 1-ethyl-3-(-3-dimethylaminopropyl) and carbodiimide/N-hydroxysuccinimide (EDC-NHS) solution (0.4 M–0.1 M). This allowed for the activation of the -COOH terminal group of MHDA. Following the washing with DI water, the samples were exposed for 1 h to the anti-*L. pneumophila* pAb at 100 µg/mL in 1× PBS that bind through their amine group to the activated -COOH. To saturate the unreacted -COOH groups, the chips were exposed for 1h at pH 8 in a 1M of ethanolamine solution. Following three times of washing with 1× PBS, the samples were exposed for 1 h to either *L. pneumophila* SG1 or SG5 suspensions in 1× PBS at 10^6^ CFU/mL. Finally, the samples were washed with DI water and imaged by optical microscopy to determine bacterial surface coverage.

### 2.5. Processing of Cooling Tower Water for Biosensing Experiments

For biosensing experiments, 10 mL of cooling tower (CT) water from the Université de Sherbrooke was filtered through a 0.22 µm syringe filter. The retained matter was washed in triplicate with 10 mL of DI water. Finally, the filter was backwashed using 10 mL of 0.1× PBS to collect the CT-suspended matter. The backwashed samples were spiked with *L. pneumophila* SG1 or SG5 employed for the exposure of AMP-functionalized GaAs/AlGaAs chips designed for capturing bacteria.

### 2.6. Optical Microscopy Analysis

The surface density of bacteria immobilized on GaAs bulk samples was determined by optical microscopy imaging (Zeiss Instruments, Inc., Oberkochen, Germany). The images were captured under 200× magnification from at least three different regions of individual samples to show the distribution bacteria (Figure S1, see Supplementary Material). The size of bacteria was confirmed by a high-magnification image (Figure S2). The experiments were repeated three times for statistical analysis. ImageJ software was used to subtract particles and enumerate bacterial surface coverage.

### 2.7. PCR Measurements

DNA of *L. pneumophila* SG1 and SG5 were extracted from the AMP-functionalized GaAs biochip for conducting PCR experiments. AMP-coated GaAs wafers were exposed to 10^6^ CFU/mL of *L. pneumophila* SG1 and SG5 for 1 h. The bacteria captured by GaAs were heated for 80 °C for 30 min with the quick DNA-extract solution kit. Thereafter, the DNA containing supernatants were centrifuged for 5 min at 10,000 RPM, and 5 µL of solution was taken for the PCR reaction. Standard real-time PCR protocol was followed for conducting PCR reactions (35 cycles) using the qPCR Illumina machine [49]. The *mip* gene-specific forward primer (5′-TTGTCTTATAGCATTGGTGCCG-3′) and reverse primer (5′-CCAATTGAGCGCCACTCATAG-3′) were used for the reactions. The PCR fluorescence value at 35 cycles were considered to compare the variation.

### 2.8. Photoluminescence Measurements

The detection of bacteria was carried out at room temperature using a quantum semiconductor photonic biosensing reader (QSPB-1) described previously [43,45]. The reference and bacteria-coated biochips were irradiated with a light-emitting diode (LED) at 660 nm. Photocorrosion was monitored by measuring photoluminescence (PL) of intermittently irradiated biochips (5 s irradiation in 20 s total period) with an intensity-homogenized beam delivering power density of ~17 mW/cm^2^ to the biochip surface. All experiments were repeated at least three times for statistical analysis. The experiments carried out in a 0.1× PBS solution (without bacteria) were used to obtain the reference measurements.

### 2.9. Statistical Analysis

Statistical analyses were performed using Graphpad Prism™ (Graphpad Software, San Diego, CA, USA). Bacterial capture and RT-PCR data were evaluated by two-way analysis of variance (ANOVA) followed by post-hoc analysis using Tukey’s multiple comparison test. For bacterial capture, bacteria and AMP coating were independent variables. Serogroup and AMP coating were independent variables for the quantitative measurement of the *mip* gene by RT-PCR. Capture efficiency of *L. pneumophila* SG1 versus SG5 was tested using an unpaired Student’s *t*-test. For biosensor experiments, peak PL values were compared to no-bacteria controls using 1-way ANOVA followed by Tukey’s (pristine water) or Dunnett’s (cooling tower water) multiple comparison tests. In all analyses, a *p* < 0.05 was considered statistically different.

## 3. Results and Discussion

### 3.1. Functionalization of GaAs/AlGaAs Biosensors

The immobilization of AMPs on GaAs surface was verified using FTIR-absorbance measurements followed by the same procedure as reported by Islam, Hassen, Tayabali, and Dubowski [40]. The absorbance band at 1605 cm^−1^ that is well known for C=O stretching could be assigned to the amide II [50,51] (see Table S1 for FTIR absorbance bands reported in literature). The absorbance bands at 1655 cm^−1^ and 1734 cm^−1^ are assigned to the amide I and amide II, respectively [52,53], and suggest the characteristic presence of a helical conformation of the surface-conjugated peptide [54,55,56]. Similarly, the peptide immobilized through the C-terminus and with free N-terminus shows characteristic peaks at 1655 cm^−1^ for the peptide [56,57]. The band observed at 1734 cm^−1^ is the C=O stretching of lateral chain functions and of some hydrolysed ester functions [53,55] and the absorbance band at 3218 cm^−1^ could be assigned to the amide A [57,58]. Therefore, the amide-related peaks in the FTIR spectra (1605 cm^−1^, 1655 cm^−1^, 1734 cm^−1^, and 3218 cm^−1^) confirm the AMP immobilization on the GaAs surface. Furthermore, the XPS amide-related peak at 288.08 eV observed for our samples [40] corroborates the successful immobilization of peptides, in agreement with Corrales-Ureña and colleagues [59].

### 3.2. Bacteria-Capture Efficiency by Peptide-Coated Surfaces

**Figure 2:**
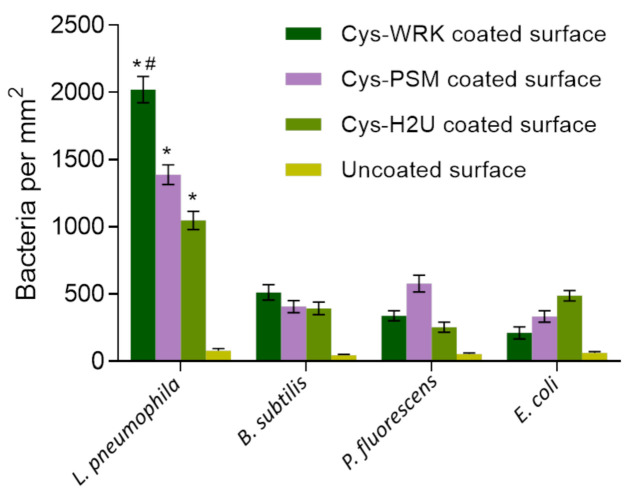
Bacterial-capture efficiency enumerated by optical microscopy following conjugation of different peptides with GaAs chips. Cysteine-modified warnericin AMP biosensor captured *L. pneumophila* 4 times more efficiently than the other investigated bacteria. Error bars represent standard error of the mean for five separate experiments. Statistical differences were measured by 2-way ANOVA followed by Tukey’s multiple comparison test with different bacteria and coatings as variables affecting capture efficiency. The asterisks (*) indicate significantly different values of *L. pneumophila* compared to reference bacteria (*p* < 0.0001). The hash (#) indicates significantly different values for *L. pneumophila* on Cys-WRK compared to other coatings (*p* < 0.05).

To evaluate the specificity of the AMPs used for *L. pneumophila* capture, a series of experiments were conducted by exposing GaAs bulk samples functionalized with Cys-WRK, Cys-PSM, or Cys-H2U to *L. pneumophila,* while negative control runs were collected for *B. subtilis*, *P. fluorescens*, and *E. coli* suspensions at 10^6^ CFU/mL. The background signal was measured by exposing bare GaAs to the investigated bacteria. The bacterial-capture efficiencies (bacteria/mm^2^) are presented in Figure 2 (examples of optical microscopy images for each case are shown in Figure S1). The average density of bacteria captured by the Cys-WRK peptide-functionalized GaAs were 2021, 338, 512, and 211 bacteria/mm^2^ for *L. pneumophila*, *P. fluorescens*, *B. subtilis*, and *E. coli*, respectively. Furthermore, Cys-WRK captured 1.5 to 2 times more *L. pneumophila* (2021 bacteria/mm^2^) compared to the Cys-H2U and Cys-PSM based biosensor architectures.

These results illustrate that the investigated peptides bind *L. pneumophila* more efficiently than the other investigated bacteria, consistent with earlier reports [60,61,62]. Furthermore, the Cys-WRK AMP has a significantly higher binding affinity towards *L. pneumophila* than the other investigated peptides. This superior performance in capturing *L. pneumophila* could be related to the lipid composition of the *L. pneumophila* membrane. For instance, it has been reported by Verdon, et al. [63] that the presence of branched-chain fatty acids, such as C15:0, C 16:0, and C 17:0 on the surface of *L.* *pneumophila* is associated with bacterial specificity of warnericin RK AMP. Another study has suggested that the high proportion (30%) of phosphatidylcholine, also known as lecithin, on the outer membrane of *Legionella* leads to a specific interaction with the Cys-WRK peptide [64,65]. Marchand, Augenstreich, Loiseau, Verdon, Lecomte, and Berjeaud [61] reported that two specific amino acids present in the Cys-WRK sequence at the 4th and 17th position are also associated with the specific interaction between peptide and *L. pneumophila.* Nevertheless, more study is required to further elucidate the reasons for the enhanced specific interaction between the Cys-WRK peptide and *L. pneumophila* bacteria.

### 3.3. Reactivity of L. pneumophila pAb against L. pneumophila SG1 and SG5

**Figure 3:**
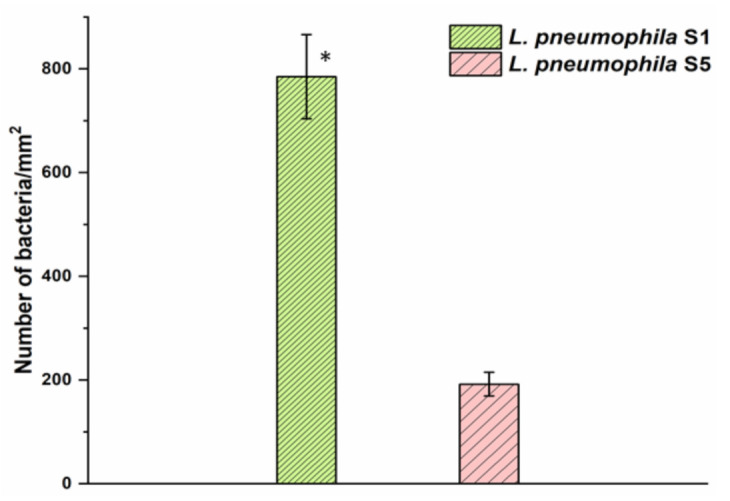
The capture efficiency of *L. pneumophila* SG1 and SG5 with pAb-functionalized GaAs surface. Error bars represent standard error of the mean from three separate experiments. The asterisk indicates significantly different values compared to the reference as determined by the Student’s test (n = 3, *p* < 0.05).

Figure 3 represents the surface coverage of the pAb-functionalized GaAs chips showing the number of captured *Legionella* at 785/mm^2^ (dense pattern) and 192/mm^2^ (light pattern) corresponding to SG1 and SG5, respectively. Thus, at the same test concentration (10^6^ CFU/mL) of both *Legionella* serogroups, the binding efficiency of the pAb was approximately four times greater for SG1 compared to the SG5 serogroup. The higher affinity of the pAb towards *L. pneumophila* SG1 could be due to the fact that the preparation of these ligands was based on the interaction with the whole cell of that strain [48].

However, it is important to note that the *L. pneumophila* strains used here were isolated from different environments and demonstrated distinct genetic backgrounds [66]. The lipopolysaccharide (LPS) characteristic and phenotype of the strains used to produce these pAbs could explain the increased capture efficiency observed with *L. pneumophila* SG1 strains.

It is worth mentioning that working with large concentrations of bacteria permitted statistical validation of the results by microscopic enumeration of bacteria. In the case of weakly concentrated bacterial suspensions, the enumeration of bacteria would carry excessively large errors as the capture efficiency of the biofunctionalized chips is below 1%. Thus, we have not attempted to conduct macroscopic enumeration of bacteria for suspension at ≤100 CFU/mL as discussed later in this report.

### 3.4. Reactivity of L. pneumophila SG1 and SG5 against Different Peptides

**Figure 4:**
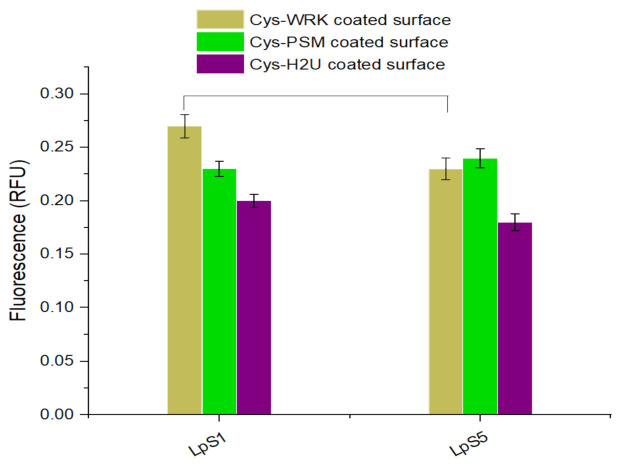
Quantitative PCR results (relative fluorescence units, RFU) for *L. pneumophila* SG1 and *L. pneumophila* SG5 captured by peptide-functionalized biosensors. Error bars represent standard error of the mean from three separate experiments. Statistical differences were measured by 2-way ANOVA followed by Tukey’s multiple comparison test with *L. pneumophila* serogroups and coatings as variables affecting amplification of the *mip* gene. The horizontal lines between bars indicate significantly different values between serogroups (*p* < 0.05).

The reactivity of *L. pneumophila* SG1 and SG5 against the AMP-coated GaAs surface was tested using PCR. The PCR fluorescence data presented in Figure 4 shows a significant difference between fluorescence intensities corresponding to *L. pneumophila* SG1 and SG5 for the Cys-WRK-coated surface while insignificant differences were observed for other peptides (see Figure S3 for the related real-time PCR amplification plots). The results suggest that the Cys-WRK-coated GaAs offers a certain level of specificity for selective capture of *L. pneumophila* SG1. However, as shown in Figure 2, some other microbes could also be bound by this peptide. Therefore, the selectivity offered by Cys-WRK AMP is not sufficient in designing a biosensor highly specific to *L. pneumophila* SG1.

### 3.5. Selective Detection of L. pneumophila SG1 and SG5 Using AMP-Ab Sandwich Technique

**Figure 5:**
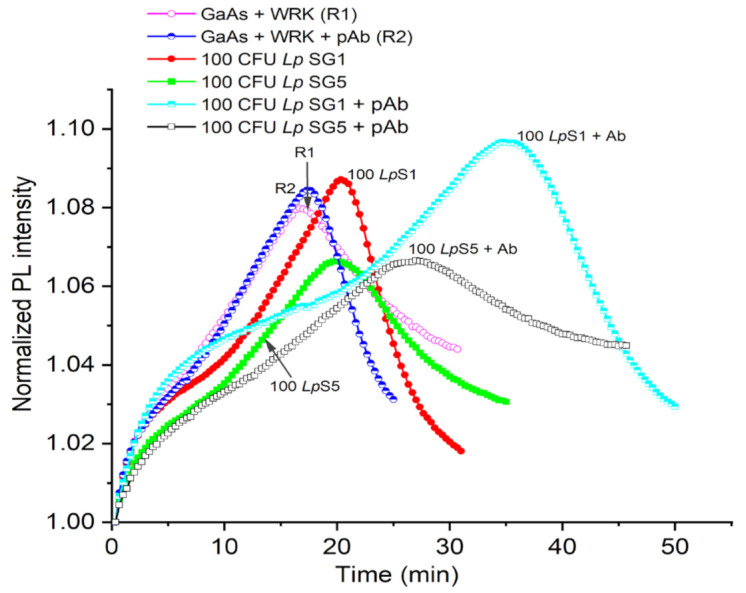
Normalized PL intensity of AMP-functionalized GaAs/AlGaAs DIP biochips (wafer D3422) exposed to bacteria in 1× PBS. The open circle (R1) and semicircle (R2) plots represent reference without exposing to bacteria. The red full circle and green square plots represent the exposure to 100 CFU/mL of *L. pneumophila* SG1 and SG5, respectively. The black open square and cyan semisquare plots represent the exposure to 100 CFU/mL of pAb-decorated *L. pneumophila* SG1 and SG5, respectively.

The utilization of DIP GaAs/AlGaAs biosensors functionalized with Cys-WRK AMP peptide to capture *L. pneumophila* SG1 and SG5 and use of pAb to detect them is summarized in Figure 5 and Table 2. We show examples of the biosensing runs for *L. pneumophila* SG1 (red full circles) and SG5 (green full squares) bacterial suspensions at 100 CFU/mL. The reference runs in this figure were collected for GaAs/AlGaAs functionalized with Cys-WRK (plot R1, purple open circles) and after the exposure of GaAs/AlGaAs functionalized with Cys-WRK to anti-*L. pneumophila* SG1 pAb (plot R2, blue semi-circles). As discussed by Aziziyan and colleagues [21], the time-dependent positions of PL intensity maxima correspond to the front passing through the GaAs/AlGaAs interface, and thus, it is a measure of the rate of photocorrosion. The identical positions of PL maxima (~20 min) revealed for SG1 and SG5 illustrate the inability of a biosensor to distinguish the investigated strains. However, the capture of bacteria from 100 CFU/mL suspensions of *L. pneumophila* SG1 and SG5, followed by the incubation in the pAb showed PL maxima occurring at 36 min (cyan semi-squares) and 25 min (black open squares), respectively. This significant delay of the PL maximum for the pAb-decorated *L. pneumophila* SG1 (~16 min) demonstrates that the sensitivity of DIP PL biosensors is enhanced after decorating bacteria with the pAb. We attribute this to the interaction of the pAb with AMP-captured *L. pneumophila* SG1 and transfer of the additional charge from the negatively charged pAb [67] to the biochip surface.

The influence of the pAb on the photocorrosion rate of GaAs/AlGaAs chips was investigated in separate experiments concerning DIP runs collected for a biochip functionalized with MHDA self-assembled monolayer and for a biochip functionalized with pAb after the -COOH group of MHDA was activated with the EDC/NHS procedure (see Figure S4). A significant delay of the PL maximum position was observed for the MHDA-pAb architecture compared to the PL maximum observed for the MHDA-only-functionalized biochip. This behaviour is consistent with the flow of a negative charge to the biochip surface also observed for other GaAs/AlGaAs nanoheterostructures [23,42,43,45].

A greater delay of the PL maximum observed in Figure 5 for *L. pneumophila* SG1 compared to SG5 (~11 min) is consistent with the relatively greater selectivity of the pAb towards *L. pneumophila* SG1 (see Figure 3). Furthermore, the exposure of a reference sample to the pAb alone did not show a significant change in the delay of a PL-intensity maximum (blue semicircles), which is related to the weak pAb–AMP interaction [68]. We also observed similar response of the GaAs/AlGaAs biochips to 50 CFU/mL of *L. pneumophila* SG1 and SG5 decorated with pAbs, as shown in Table 1 (see Figure S5 in Supplementary Information). These results are consistent with the observation that decorating *L. pneumophila* SG1 with negatively charged SDS molecules significantly enhanced the sensitivity of DIP biosensors as reported in [23].

**Figure 6:**
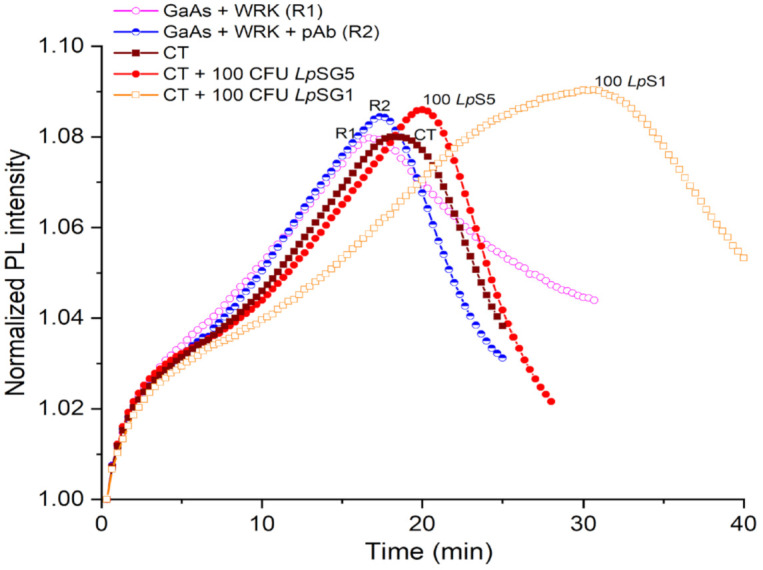
Normalized PL intensity of AMP-functionalized GaAs/AlGaAs DIP biochips (wafer D3422) exposed to CTW spiked with *L. pneumophila* at 100 CFU/mL. The purple open circle (R1) and blue semicircle (R2) plots represent reference without exposing to bacteria. The red full circle and brown square plots represent the biochip response to pAb-decorated *L. pneumophila* SG5 and SG1, respectively.

Examples of biosensing runs of DIP GaAs/AlGaAs biosensors responding to *L. pneumophila* captured from CTW suspensions with *L. pneumophila* SG1 and SG5 at 100 CFU/mL (see Section 2.5) are presented in Figure 6 and Table 1. It can be seen that PL maxima for *L. pneumophila* SG5 (red full circles) and SG1 (brown open squares) occur at 22 min and 31 min, respectively. The significantly greater delayed PL maximum for *L. pneumophila* SG1 compared to *L. pneumophila* SG5 could be attributed to the selectivity generated through pAb conjugation. We note that the weaker delay of PL maxima observed for *L. pneumophila* SG1 and SG5 in CTW compared to pristine conditions (Figure 5) might be related to the presence of ionic species in CTW that affect the capture efficiency of bacteria by AMP-functionalized GaAs/AlGaAs chips. Under these conditions, we were not able to detect *L. pneumophila* SG1 at 50 CFU/mL, and thus, detection at 100 CFU/mL determines the current limit of detection (LOD). The DIP-biosensor technology has been investigated for detection of different bacteria, including *E. coli*, *Bacillus* sp., and *L. pneumophila* [23,39,44], with typical LOD at 10^3^ CFU/mL. Therefore, detection of *L.* *pneumophila* SG1 at 100 CFU/mL represents a significant step towards development of a field-operating DIP biosensor that is expected to deliver enhanced biosensing based on the introduction of filtration and preconcentration techniques of water samples originating from different sources [69].

The application of Cys-WRK AMP for functionalization of GaAs-based DIP biosensors permitted the elimination of the 20-h step required for (a) formation of MHDA SAM and (b) EDC/NHS activation of the -COOH group for binding with pAb through their amine group. Consequently, the ~15 nm long bacteria-binding architecture was replaced with a significantly shorter, ~2 nm long ligand fabricated in less than three hours. While the elimination of the extra EDC/NHS biofunctionalization step contributes to the more consistent data collection, the short chain ligands support more efficient charge transfer between pAb-decorated bacteria and the surface of a biosensor. Thus, the short-chain AMP architectures modified with the sandwich-biosensing technique is highly attractive for rapid, sensitive, and specific detection of pathogenic bacteria using charge-sensing devices, such as DIP biosensors.

## 4. Conclusions

We have investigated an innovative concept of an AMP–pAb-sandwich architecture for the construction of a DIP GaAs/AlGaAs biosensor and selective detection of *L. pneumophila* SG1 and SG5. The biosensor was first functionalized with Cys-AMPs and incubated with *L. pneumophila*. This was followed by decorating bacteria with anti-*L. pneumophila* pAb. Our results demonstrated the detection sensitivity as low as 50 CFU/mL for bacterial suspensions in pristine conditions, and 100 CFU/mL in samples originating from cooling tower water. The proposed method enhanced the sensitivity and specificity of the biosensor and allowed selective detection of *L. pneumophila* SG1 in both pristine and industrial water conditions. These results are attractive for the development of quasi-continuous monitoring of the water environment for the presence of bacteria with DIP biosensors comprising stacks of GaAs/AlGaAs bilayers designed to deliver a series of data with a single device. The results have potential to be applied to the development of other biosensing devices.

## Figures and Tables

**Table 1:** Immunosensors proposed for the detection of *L. pneumophila*.

Detection Technique	Substrate for Immobilization	Bioreceptors	Detection Source	Time for Result	Range of Detection (CFU/mL)	Limit of Detection (CFU/mL)	Reference
SPR	Au	mAb	PBS	2 h 20 min	10^2^–10^9^	10^2^	[24]
EIS	Au	mAb	PBS	-	10^1^–10^8^	10^1^	[12]
Microelectrode array	Si	Antibody	PBS	-	10^5^–10^8^	10^5^	[25]
EIS	Au	Antibody	PBS	-	2 × 10^1^–2 × 10^5^	2 × 10^2^	[11]
Amperometric sensor	Carbon	pAb	PBS	3 h	10^4^–10^6^	10^4^	[26]
SPR	Au	mAb	PBS	-	10^1^–10^4^	10^1^	[18]
SPR	Au	pAb	PBS	30 min	10^3^–10^6^	10^3^	[13]
Colorimetric	Gold nanoparticles	Nucleic Acid	DI water	60 min	-	124	[15]
DIP	GaAs/AlGaAs	pAb	PBS	42 min	10^4^–10^6^	10^4^	[21]
DIP	GaAs/AlGaAs	pAb/SDS	PBS	70 min	10^2^–10^6^	10^3^	[23]

Ab: monoclonal antibody, pAb: polyclonal antibody.

**Table 2:** PL maxima obtained for the reference (PBS) run and after the exposure of *L. pneumophila* (all experiments repeated for at least 3 times).

Bacteria and Reference	PL Maxima (Minutes)	Significantly Different vs. Control (*p* Value)
**Pristine Condition**		
GaAs + Cys-WRK	16 ± 1.12	Control
GaAs + Cys-WRK + Anti *Lp* pAb	17.50 ± 1.18	No
GaAs + Cys-WRK + 100 CFU/mL of *Lp*SG1	21.05 ± 1.5	No
GaAs + Cys-WRK + 100 CFU/mL of *Lp*SG5	19.23 ± 1.2	No
GaAs + Cys-WRK + 50 CFU/mL of *Lp*SG1 + Anti *Lp*SG1 pAb-decorated bacteria	27.83 ± 2	Yes (*p* < 0.0001)
GaAs + Cys-WRK + 50 CFU/mL of *Lp*SG5 + Anti *Lp*SG1 pAb-decorated bacteria	21 ± 1.14	No
GaAs + Cys-WRK+100 CFU/mL of *Lp*SG1 + Anti *Lp*SG1 pAb-decorated bacteria	36.2 ± 2.1	Yes (*p* < 0.0001)
GaAs + Cys-WRK + 100 CFU/mL of *Lp*SG5 + Anti *Lp*SG1 pAb-decorated bacteria	25.75 ± 1.16	Yes (*p* < 0.0019)
**Cooling Tower Condition**		
Cooling tower water (3IT)	18.37 ± 1.5	Control
GaAs + Cys-WRK + Cooling tower water + 100 CFU/mL of *Lp*SG5 + Anti *Lp*SG1 pAb	22.20 ± 2	No
GaAs + Cys-WRK + Cooling tower water + 100 CFU/mL of *Lp*SG1 + Anti *Lp*SG1 pAb	31.5 ± 2	Yes (*p* < 0.0043)

## Supplementary Materials

The following supporting information can be downloaded at: https://www.mdpi.com/article/10.3390/bios12020105/s1. Figure S1: Representative optical micrographs of bacteria on uncoated and AMP-functionalized GaAs surface, Figure S2: An example of optical micrograph for determining *L. pneumophila*, Figure S3: Real-time PCR amplification curves for *L. pneumophila* SG1 and SG5 captured by different peptides, Figure S4: Normalized PL intensity runs of MHDA functionalized GaAs/AlGaAs DIP biochip and following its exposure to pAb, Figure S5: Normalized PL intensity of AMP functionalized GaAs/AlGaAs DIP biochips exposed to *L. pneumophila* SG1 and SG5 at 50 CFU/mL and decorated with pAb, Table S1: FTIR absorbance bands corresponding to the assigned functional groups.
