# Supplementary material for: Selective Detection of Legionella pneumophila Serogroup 1 and 5 with a Digital Photocorrosion Biosensor Using Antimicrobial Peptide-Antibody Sandwich Strategy
Source: Biosensors (Basel). 2022 Feb 9;12(2):105. doi: 10.3390/bios12020105 (PMC8869675; doi:10.3390/bios12020105)
Supplement: Supplementary file 1 [file biosensors-12-00105-s001.zip › biosensors-1519013-supplementary.pdf]

Supporting Information

Selective Detection of *Legionella pneumophila* Serogroup 1 and 5 with a Digital Photocorrosion Biosensor Using Antimicrobial Peptide-Antibody Sandwich Strategy

M. Amirul Islam[1], Walid M. Hassen[1], Ishika Ishika[1], Azam F. Tayabali[2], Jan J. Dubowski[1*]

[1]Interdisciplinary Institute for Technological Innovation (3IT), CNRS UMI-3463, Laboratory for Quantum Semiconductors and Photon-based BioNanotechnology, Department of Electrical and Computer Engineering, Université de Sherbrooke, 3000, boul. de l'Université, Sherbrooke, Québec J1K 0A5, Canada; mohammed.amirul.islam@usherbrooke.ca (M.A.I.); mohamed.walid.hassen@usherbrooke.ca (M.W.H.); Ishika.Ishika@usherbrooke.ca (I.I.)

[2]Biotechnology Laboratory, Environmental Health Science and Research Bureau, Healthy Environments and Consumer Safety Branch, Environmental Health Centre, Health Canada, Ottawa, Ontario K1A 0K9, Canada; azam.tayabali@hc-sc.gc.ca (A.T.)

[*]Corresponding author: jan.j.dubowski@usherbrooke.ca (J.J.D.)

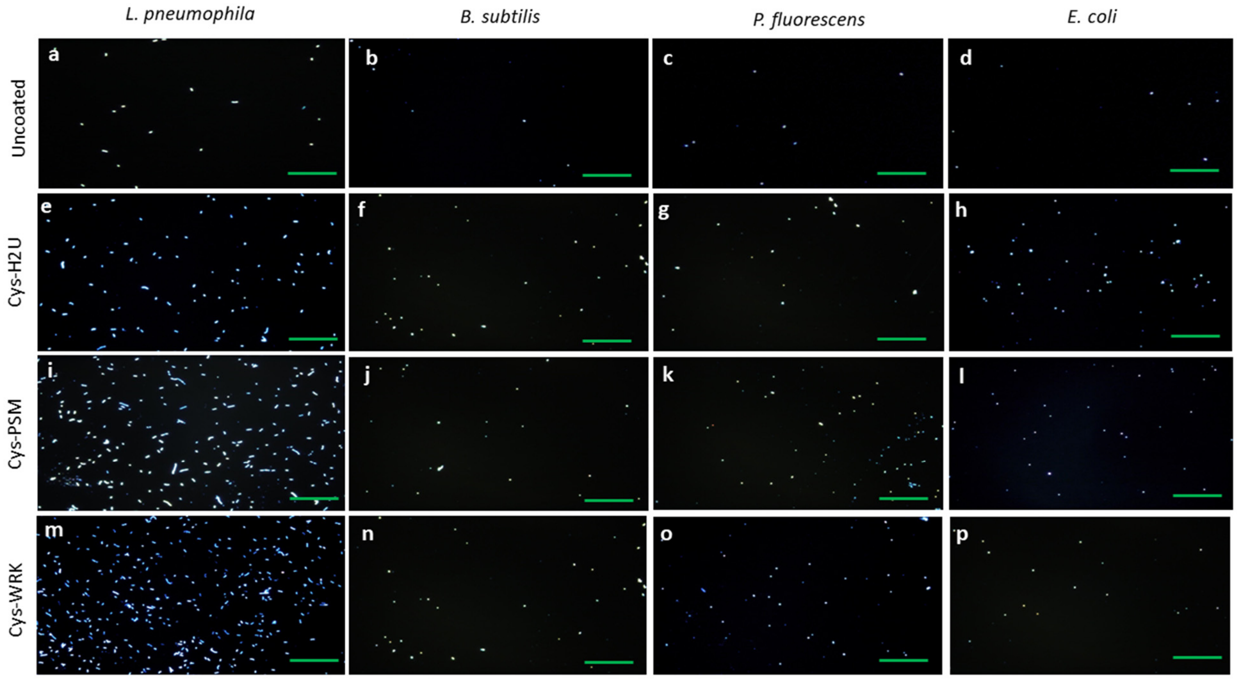

Figure S1. Representative optical micrographs of different bacteria on uncoated and different AMP (50 μg/mL) functionalized surfaces of GaAs. The scale bar represents 100 μm.

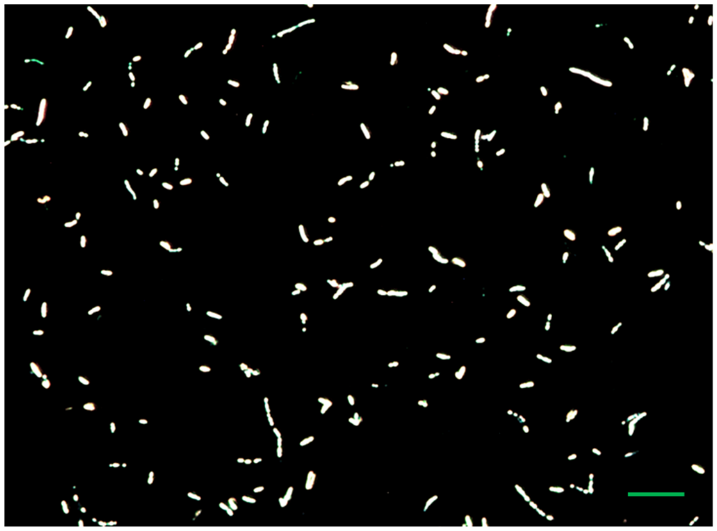

Figure S2. An example of optical micrograph for determining *L. pneumophila*. The scale bar represents 15 μm.

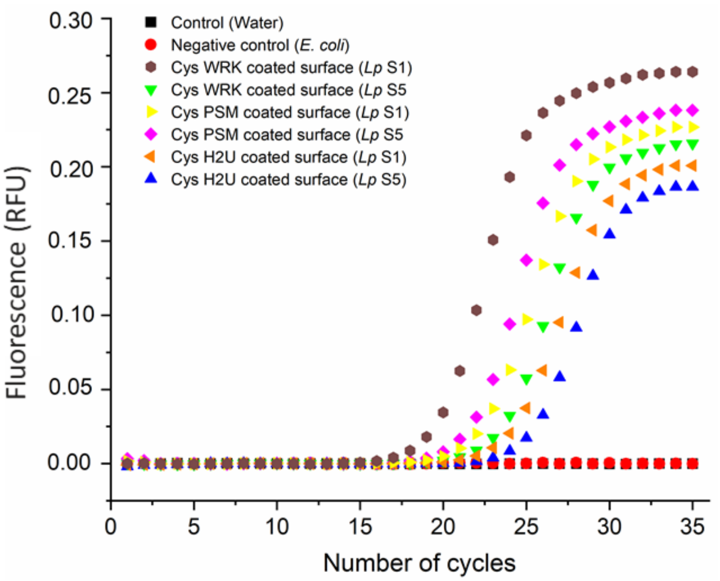

Figure S3. The real-time PCR amplification curves (relative fluorescence units, RFU) for *L. pneumophila* SG1 and *L. pneumophila* SG5 captured by different peptide-functionalized GaAs samples.

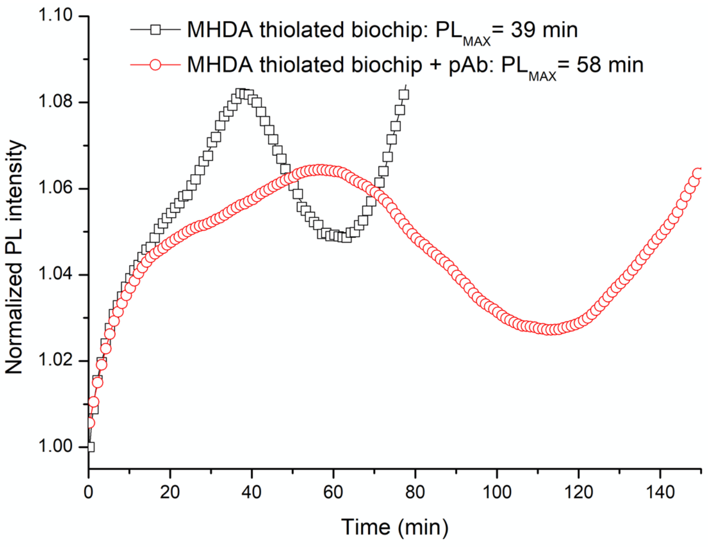

Figure S4. Examples of DIP runs for MHDA thiolated GaAs/AlGaAs biochip before (squares) and after (circles) pAb attachment. The figure presents example of PL data for the GaAs/AlGaAs biochips following a 20-hours functionalization with 1mM mercaptohexadecanoic acid (MHDA) thiol and after Ab attachment (incubation in 100 μg/mL Ab solution for 1-hour). The MHDA-based architecture was designed to conduct control experiment, however, data presented in the main part of the manuscript were collected using AMP-based architectures.

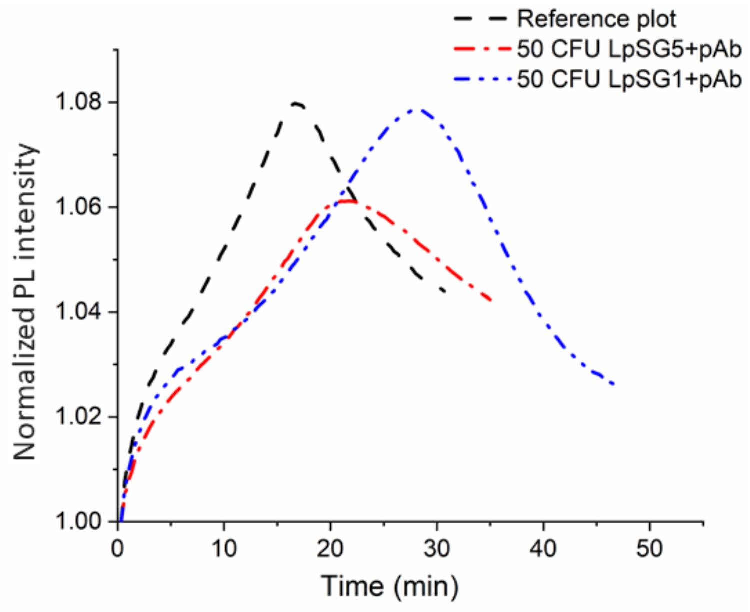

Figure S5. Normalized PL intensity of AMP functionalized GaAs/AlGaAs DIP biochips (wafer D3422) exposed to *L. pneumophila* (SG1 and SG5) at 50 CFU/mL and decorated with pAb.

Table S1. FTIR absorbance bands corresponding to the assigned functional groups

Absorbance Bands (cm^{-1})	Corresponding Bonds	Reference
1605	Amide II	[1,2]
1655	Amide I bond (C=O stretching)	[3,4]
1738	Amide II, C=O stretching lateral chain function	[5,6]
3218	Amide A	[7,8]

References

1. Choo, E.S.G.; Tang, X.; Sheng, Y.; Shuter, B.; Xue, J. Controlled loading of superparamagnetic nanoparticles in fluorescent nanogels as effective T 2-weighted MRI contrast agents. *Journal of Materials Chemistry* **2011**, *21*, 2310–2319
2. Yuen, S.-N.; Choi, S.-M.; Phillips, D.L.; Ma, C.-Y. Raman and FTIR spectroscopic study of carboxymethylated non-starch polysaccharides. *Food Chem.* **2009**, *114*, 1091–1098
3. Ami, D.; Posteri, R.; Mereghetti, P.; Porro, D.; Doglia, S.M.; Branduardi, P. Fourier transform infrared spectroscopy as a method to study lipid accumulation in oleaginous yeasts. *Biotechnology for biofuels* **2014**, *7*, 12
4. Barbosa, M.; Vale, N.; Costa, F.M.; Martins, M.C.L.; Gomes, P. Tethering antimicrobial peptides onto chitosan: Optimization of azide-alkyne "click" reaction conditions. *Carbohydrate polymers* **2017**, *165*, 384–393
5. Humblot, V.; Yala, J.-F.; Thebault, P.; Boukerma, K.; Héquet, A.; Berjeaud, J.-M.; Pradier, C.-M. The antibacterial activity of Magainin I immobilized onto mixed thiols Self-Assembled Monolayers. *Biomaterials* **2009**, *30*, 3503–3512
6. Doiron, K.; Beaulieu, L.; St-Louis, R.; Lemarchand, K. Reduction of bacterial biofilm formation using marine natural antimicrobial peptides. *Colloids Surf. B. Biointerfaces* **2018**, *167*, 524–530
7. Dinesh, B.; Squillaci, M.A.; Ménard-Moyon, C.; Samorì, P.; Bianco, A. Self-assembly of diphenylalanine backbone homologues and their combination with functionalized carbon nanotubes. *Nanoscale* **2015**, *7*, 15873–15879
8. Jeevithan, E.; Bao, B.; Bu, Y.; Zhou, Y.; Zhao, Q.; Wu, W. Type II collagen and gelatin from silvertip shark (Carcharhinus albimarginatus) cartilage: Isolation, purification, physicochemical and antioxidant properties. *Mar. Drugs* **2014**, *12*, 3852–3873
